# Monthly Summary

**Published:** 1876-10

**Authors:** 


### MONTHLY SUMMARY.

---

*The Comparative Effects of Ether and Chloroform.*—From a recent number of the *British Medical Journal* we learn that Professor Schiff made a verbal communication to the Medico-Physical Society of Florence, on March 1st, in which he related the results of upwards of five thousand experiments on the differences between anæsthesia produced by chloroform and that produced by ether. With both ether and chloroform, paralysis of conscious sensation; paralysis of the movement of voluntary muscles; paralysis of respiration, circulation, and, finally paralysis of the heart and the vasomotor nerves, occur. Respiratory paralysis is produced by ether when circulation and blood-pressure remain within the limits compatible with life. Sometimes the vascular pressure increases, sometimes it decreases, but it is always sufficiently high to allow the exchange of the carbonic acid gas with the oxygen of the atmosphere. Vascular succeeds respiratory paralysis when ether is administered. The reverse takes place with chloroform. Frequently an amount of this anæsthetic agent which would not be sufficient to produce respiratory, may suffice to bring on vascular paralysis. Under these conditions, and when vascular paralysis lasts under thirty seconds, artificial respiration is useless, because there is no longer any exchange of gases, the blood pressure being diminished. The cessation of respiration, therefore is not the most dangerous moment to animal life, when etherization is employed, whilst it may be so with chloroformization, because sometimes it is possible to produce some automatic respiratory movements, but nevertheless respiration ceases immediately, and the animal dies. With etherization, on the contrary, when some automatic inspirations are obtained, it may be taken as certain that respiration will continue and that the animal will live. Professor Schiff affirms that in the present state of science, there are no means which will show us how to recognize, so as to prevent them, the tendencies which may cause death in some animals after the first inhalations of chloroform, before having produced true anæsthesia. The reverse occurs with ether; so that it may be said that in the present state of knowledge the surgeon is responsible for the death of the individual by etherization; whilst he is not responsible when death occurs during chloroformization. Professor Schiff, therefore, deduces from these facts the following conclusions: 1. The phenomena relating to the paralysis of sensibility and movement are common to both ether and chloroform. 2. The two other orders of phenomena, that is to say, those relating to vascular and respiratory paralysis, often show themselves in inverse order with reference to these two agents. 3. With chloroform, however, either the one or the other of these two paralyses may first show itself, involving great danger to the animal if the vascular phenomena be the first to make their appearance. Therefore the use of chloroform should be rejected and ether only be used.—*Med. and Surg. Reporter.*

---

*Caution in the use of Chloral.*—At the last Annual Meeting of the Association of Superintendents of American Asylums for the Insane, held in Philadelphia, the subject of chloral came in for a share of discussion. As physicians attached to lunatic asylums have probably more occasion to observe the effects of the drug than practitioners otherwise situated, the relation of their experience cannot fail to command attention. The remarks given below, are taken from the report published in the *Medical Record.* Dr. T. H. R. Smith, of Lunatic Asylum No. 1, Fulton, Mo., introduced the subject of the use of chloral, which he considered a highly important one. He thought much injury was done by the injudicious use of this drug, and regarded it as a remedy which ought to be used with the greatest care, and the results closely watched. He believed that frequently the results of the chloral were mistaken for the regular progress of the disease, and that sometimes it had caused death. In chronic cases of insanity he regarded chloral as a very unsafe remedy. Also in the cases of aged people.

Dr. George C. Catlett, of Lunatic Asylum No. 2, at St. Joseph, Missouri, said that he knew of three well-authenticated cases of death from the administration of ordinary doses of chloral as a sedative in cases of delirium from intemperance.

Dr. C. F. MacDonald, of the State Lunatic Asylum for Insane Criminals, at Auburn, N. Y., remarked that on an occasion an attendant gave a patient two drachms of chloral by mistake. The patient suffered from acute mania, and soon after taking the dose fell into a heavy sleep, and afterwards awoke calmer and quieter, and progressed to a complete recovery.

Dr. A. E. MacDonald, of the City Asylum for the Insane, at Ward's Island New York, considered chloral a valuable remedy and has used it a great deal in his practice. He had never heard of a death said to be from chloral, where the post mortem examination did not reveal other causes for death.

Dr. Kirkbride said that his sad experience with chloral had been that sometimes patients died unexpectedly to him, and he would not like his medical friends to administer it to him under any circumstances.

Dr. Wm. M. Compton, of the Lunatic Asylum, Jackson Miss., said that there were several noisy patients in his institution who had taken doses of from 20 to 30 grains of chloral every evening for years, merely to quiet them and produce sleep, and he had never seen any bad effects.—*Druggists' Circular.*

---

*Composition of the Human Body.*—A complete analysis of a man recently made by Dr. Lancaster, of London, has been described by him in a chemical lecture. The body operated upon weighed 158·4 lbs, and the lecturer exhibited upon the platform 23·1 lbs. carbon, 2·2 lbs. lime, 22·3 ounces phosphorus, and about one ounce each of sodium, iron, potassium, magnesium and silicon. Dr. Lancaster apologized for not exhibiting 5585 cubic feet of oxygen, weighing 121 lbs., 105,000 cubic feet of hydrogen, weighing 15·4 lbs., and 52 cubic feet of nitrogen likewise obtained from the body, on account of their great bulk. All of these elements combine into the following: 121 lbs. water, 16·5 lbs. gelatine, 52 lbs fat, 8·8 lbs. fibrin and albumen, 7.7 lbs. phosphates of lime and other mineral substances.—*American Gaslight Journal.*

*The Blue Line in Lead Poisoning.*—In order to distinguish the gingival line caused by lead from other discolorations on the gums which may resemble it, M. Cras excises the edge of the gum, and examines it under the microscope. He finds that the lead-line does not result from a tattooing of the gums by some metallic particles having penetrated into the epithelial cells or more deeply. It is due to transformation of a soluble salt of lead into a sulphide precipitated in the capillaries by the retarded circulation in the gums, owing to the vicinity of the putrid detritus surrounding the neck of the teeth. It is then an injection of the capillaries by the sulphide of lead. M. Cras supposes that analogous vascular obliterations in the intestines are, perhaps, the cause of saturnine colic.

---

*Toothache Remedy.*—Mr. C. A. Guild writes "In last week's issue you quote Dr. Lardier on collodion for toothache. I have found collodion mixed with enough carbolic acid to form a jelly-like mass to be an excellent remedy for toothache. About equal parts will form a "stiff" jelly, which may be taken on the end of a pine stick and placed in the cavity of the aching tooth. The pain will be relieved almost instantly if it depends on an exposed nerve. I have found this the most reliable and convenient remedy I ever tried."—*Clinic.*

---

*Women's Sphere.*—Mrs. E. Pfeiffer Stone, M. D., writes as follows to the *Pacific Medical and Surgical Journal,* in reply to a protest against the admission of females into the State Medical Society: "When we look at women revolving in her sphere within a hundred years in England, we behold her liable to be led like cattle attached to the halter, to be sold by her husband. On the continent of Europe I saw women working with wheelbarrow and crowbar on railroads, on a par with men, side by side; and to the general observer *there*, it is all right. No protest is offered, no outcry made, no account of her competition with men, or neglect of home or domestic duties, or on any other grounds. But when she attempts to enter the formerly barred gates to the fountains of knowledge, and to become a co-laborer with man in the field of mind, then comes the outcry of injured womenhood and the fear of neglected children, not considering that those poor women who work from daybreak till dark, also have families at home, under the care of a kind Providence and an old grandmother—herself having passed through the same sphere. In the beautiful City of Dresden, I have seen them hitched with the Newfoundland dog, hauling coals through the streets, and then, if required, carrying the coals in a bushel basket up two or three flights of stairs. In this she was worse off than the dog. Pity for the beautiful dog was often elicited, but seldom for the poor women, as this is *her sphere.*

"Had our beneficent Creator intended such narrow limits for her sphere, He never would have provided her with the thinking apparatus she possesses, even with and like unto the male. Dr. Crane knows, as a physiological student, as well as I do, that the difference in the male and female brain is only quantitatively, and that the size of women's head is in harmony with the rest of her physical stature; but to recompense her for the lesser quantity, her brain is qualitatively superior, as the fibres of the cineritious substance and medullary matter are so much the finer. And not this alone, but in the formation of the female brain we find the higher and nobler organs *expanded,* and the base small; whereas, the reverse is the case in the male brain. The convolutions are not so broad and deep in the former as in man's, but are the more numerous and diversified; and herein she is again recompensed. What holds good mentally holds good physically, as the fibres of her muscular tissue are of finer texture, and hence capable of greater endurance. May I ask Dr. C. where he finds the proof of his assertion of woman's disqualifications as physician, if not in the brain? As to physical qualifications, I have not found giants among males in the profession, but a majority of delicate frames and small of stature; but this does not disqualify them so long as they are men.

"As to relative endurance physically, take the case of the nurse at the sick bed. The woman will maintain her position for weeks, if necessary, hardly changing her clothes; but the man in the same place, after one or two nights, becomes exhausted in body and in patience, and disqualified for other duties next day. But the doctor does not seem to have observed these things in his long experience.

"I hope Dr. Crane will rest in peace, and not fear that those five women admitted as members of the Medical Society will disturb the equilibrium of the medical universe. We will not resign, but cling to our rights and try to do what duty calls us to do."

---

*Nerve-stretching in Tetanus.*—The treatment of traumatic tetanus by stretching the nerve of the affected part is beginning to attract attention as a proper resort in view of the entire failure of other methods. Dr. George Callender, of St. Bartholomews, (*London Lancet,*) refers to a case recently in charge of M. Verneuial in Paris, in which a man had his hand severely crushed and showed simptoms of the disease. The median nerve at the elbow and the ulnar at the wrist, were laid bare and subjected to traction—the patient recovering completely. Dr. Callender adds: The operation is not a severe one. The nerve is exposed and is stretched, when freed from its surrounding, by traction with an ordinary vulsellum, from its central connections. No harm is likely to be sustained as a consequence. There is now abundant evidence, in the cases reported by Billroth, Nussbaum and myself, of the tolerance with which nerves submit to forcible stretching, so far as the after performance of their functions is concerned. In view of the unsatisfactory results of the treatment of traumatic tetanus as at present conducted, there is full justification for the performance of the operation as, at least, a last resource, although I should myself advocate its trial, as in the case under the care of M. Verneuil, as soon as the signs of the disease are distinctly recognized.—*Pacific Med. and Surgical Journal.*

---

*The Progress of Cadaveric Decay.*—Physicians are not unfrequently called upon to give an opinion, in the case of the discovery of corpses, of the period that has elapsed since death. The following are the rules of Dr. Caspar, given in his *Medical Jurisprudence.* The temperature is assumed to be moderate, and the body exposed to the air.

(1.) The greenish discoloration of the abdomen and the softening of the eyeballs indicate that the person has been dead from twenty-four to seventy-two hours.

(2.) After three to five days, the green discoloration has been deeper, and extended over the whole of the abdomen, including the genitals; while similar patches have begun to appear on other parts, especially the back, lower extremities, the neck, and sides of the chest.

(3.) In about eight or ten days, the greenish patches have coalesced, and changed to a reddish-green; gaseous products have become developed in the abdomen; the cornea has become concave; the sphincter ani has relaxed; and the ramifications of the subcutaneous veins can be traced on the neck, breast. and limbs.

(4.) After fourteen or twenty days, blisters have appeared on the skin, and the development of gases has become general, distending the whole body.

(5.) Lastly, after this period it is impossible to determine the date of the decease —*Med. and Surg. Reporter.*

---

*Female Doctors.*—Within the last ten years the University of Zurich has conferred the diploma of Doctor of Medicine on thirteen ladies.—*Med. Record.*

